# High frequency deep brain stimulation can mitigate the acute effects of cocaine administration on tonic dopamine levels in the rat nucleus accumbens

**DOI:** 10.3389/fnins.2023.1061578

**Published:** 2023-01-30

**Authors:** Jason Yuen, Abhinav Goyal, Aaron E. Rusheen, Abbas Z. Kouzani, Michael Berk, Jee Hyun Kim, Susannah J. Tye, Charles D. Blaha, Kevin E. Bennet, Kendall H. Lee, Hojin Shin, Yoonbae Oh

**Affiliations:** ^1^Department of Neurologic Surgery, Mayo Clinic, Rochester, MN, United States; ^2^The Institute for Mental and Physical Health and Clinical Translation (IMPACT), Barwon Health, Deakin University, Geelong, VIC, Australia; ^3^Medical Scientist Training Program, Mayo Clinic, Rochester, MN, United States; ^4^School of Engineering, Deakin University, Geelong, VIC, Australia; ^5^Queensland Brain Institute, The University of Queensland, St Lucia, QLD, Australia; ^6^Division of Engineering, Mayo Clinic, Rochester, MN, United States; ^7^Department of Biomedical Engineering, Mayo Clinic, Rochester, MN, United States

**Keywords:** substance use disorder, deep brain stimulation, nucleus accumbens, ventral tegmental area, cocaine, tonic dopamine

## Abstract

Cocaine’s addictive properties stem from its capacity to increase tonic extracellular dopamine levels in the nucleus accumbens (NAc). The ventral tegmental area (VTA) is a principal source of NAc dopamine. To investigate how high frequency stimulation (HFS) of the rodent VTA or nucleus accumbens core (NAcc) modulates the acute effects of cocaine administration on NAcc tonic dopamine levels multiple-cyclic square wave voltammetry (M-CSWV) was used. VTA HFS alone decreased NAcc tonic dopamine levels by 42%. NAcc HFS alone resulted in an initial decrease in tonic dopamine levels followed by a return to baseline. VTA or NAcc HFS following cocaine administration prevented the cocaine-induced increase in NAcc tonic dopamine. The present results suggest a possible underlying mechanism of NAc deep brain stimulation (DBS) in the treatment of substance use disorders (SUDs) and the possibility of treating SUD by abolishing dopamine release elicited by cocaine and other drugs of abuse by DBS in VTA, although further studies with chronic addiction models are required to confirm that. Furthermore, we demonstrated the use of M-CSWV can reliably measure tonic dopamine levels *in vivo* with both drug administration and DBS with minimal artifacts.

## 1. Introduction

Despite the increasing interest and resources devoted to addiction research, there has been little improvement in the clinical care and prevalence of substance use disorder (SUD) (Substance Abuse and Mental Health Services Administration [SAMHSA], 2020). In the USA alone, management and treatment of SUD costs the healthcare, welfare, and justice systems hundreds of billion dollars annually (United States Department of Health and Human Services [USDHHS], 2016; Peacock et al., 2018). Despite the development of a variety of behavioral and pharmacological therapeutic options, most SUDs patients do not get treatment, response rates are low, and relapse rates as high as 75–98% have been reported (Brandon et al., 2007; Saloner and Karthikeyan, 2015). To better manage these “treatment-refractory” patients, it is important to further our understanding in the pathophysiology of SUD. One such approach is to study the neurochemical dynamics in the central nervous system associated with drug administration, which has the potential to identify treatment targets.

Dopamine is an important neurotransmitter for neuropsychiatric diseases such as SUD, obsessive compulsive disorder, and Tourette’s syndrome (Denys et al., 2004; Oliva and Wanat, 2016; Maia and Conceicao, 2018). Therefore, controlling the release of dopamine *via* neuromodulation, as has been done for neurological diseases such as Parkinson’s disease, is potentially an effective strategy for the treatment of these pathologies. Indeed, previous attempts have been made to stimulate targets within the mesolimbic dopaminergic pathway as a means to rectify dysfunctional dopamine dynamics (Holtzheimer and Mayberg, 2011).

**FIGURE 1:**
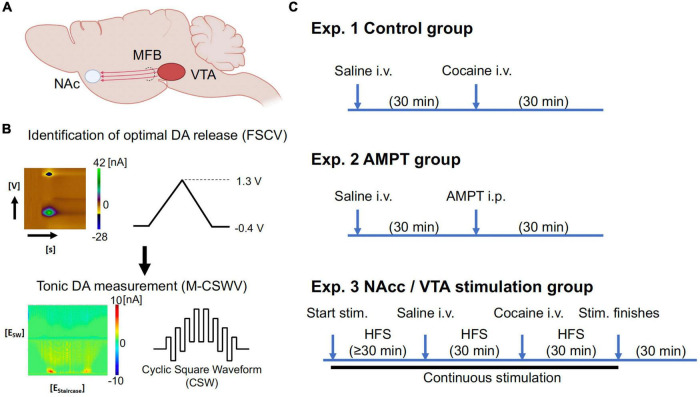
**(A)** Simplified diagram demonstrating some of the major dopaminergic projections from the VTA. **(B)** The optimal depths of electrodes in the VTA and nucleus accumbens core (NAcc) were first identified using FSCV (maximum dopamine evoked release; 60 Hz, 2 ms, 0.2 mA, 2 s duration; Supplementary Figure A1). The system was then switched to M-CSWV to record tonic dopamine levels in the NAcc. **(C)** Experimental set-up of tonic dopamine measurements. With the control and AMPT groups, no stimulation was given. Waiting time for AMPT group (250 mg/kg) was increased compared to the control group due to the different route of administration, with the expectation that i.p. injections would result in a slower onset of action than i.v. injections. The stimulation group consisted of the continuous high-frequency stimulation (130 Hz, 200 micro-sec, 0.2 mA), while saline (1 ml/kg) and cocaine (2 mg/kg) were given intravenously. Partly created with BioRender.com. *N* = 5/group (20 in total). AMPT, alpha-methyl-p-tyrosine; CFM, carbon fiber microelectrode; DA, dopamine; FSCV, fast-scan cyclic voltammetry; i.p., intraperitoneal; i.v., intravenous; stim., stimulation; M-CSWV, multiple-cyclic square wave voltammetry; MFB, medial forebrain bundle; NAc, nucleus accumbens; VTA, ventral tegmental area.

The ventral tegmental area (VTA) and substantia nigra pars compacta are major producers of dopamine in the mesolimbic dopaminergic pathway (Bjorklund and Dunnett, 2007). A major VTA projection target is the nucleus accumbens (NAc), which has been implicated in mediating important cognitive functions, such as reward and learning (see Figure 1A; Salgado and Kaplitt, 2015). In addition, over- and under-release of dopamine in the NAc are important pathophysiological conditions of neuropsychiatric diseases, such as SUD (Di Chiara, 2002). The NAc is one of the most studied deep brain stimulation (DBS) targets to modulate dopamine release in SUD in both preclinical models and human trials (Liu et al., 2008; Knapp et al., 2009; Henderson et al., 2010; Guo et al., 2013; Vassoler et al., 2013; Hamilton et al., 2015; Müller et al., 2016; Batra et al., 2017; Chen et al., 2019; Sildatke et al., 2020).

Nevertheless, the dimensions and resolution of contemporary *in vivo* measuring methods such as the use of microdialysis have limited the continuous measurement of dopamine as a useful biomarker for interventive therapy, until recently (Watson et al., 2006; Gu et al., 2015). Despite having the ability to unequivocally distinguish between different types of analytes, microdialysis probes have a relatively large dimension (∼200 μm in diameter) and the temporal resolution is of the order of ≥ 1 min (Morelli et al., 1992; Di Chiara et al., 1993; Blaha et al., 1996; Chefer et al., 2009; Oh et al., 2018; Rusheen et al., 2020). In addition, microdialysis measurements cannot be made *in situ*, requiring drawing samples from the brain for subsequent laboratory identification. The trauma caused by the probe size and the relatively low temporal resolution means this method is likely to be inadequate to detect the rapid changes in dopamine levels involved in psychopathologies in the neural structures of interest (Blaha et al., 1996; Bungay et al., 2003; Borland et al., 2005). The requirement to extract brain dialysate samples makes microdialysis unsuitable as a technique for human therapy.

We recently reported a new technique known as multiple-cyclic square wave voltammetry (M-CSWV). This method is able to measure tonic extracellular dopamine levels with unprecedented temporal resolution (10 s) and minimal trauma to the neural tissue when used in combination with carbon fiber microelectrodes (CFM) (Oh et al., 2018). This technique uses dynamic background subtraction and capacitive background current modeling to eliminate large capacitive background currents generated by the applied voltammetric waveform. This allows tonic dopamine concentrations to be measured every 10 s, something not possible with conventional fast-scan cyclic voltammetry (FSCV). Our group has previously demonstrated that M-CSWV is able to record changes in tonic dopamine levels in response to cocaine administration (Yuen et al., 2021a).

NAc DBS has shown promising results for the treatment of SUD (Liu et al., 2008; Knapp et al., 2009; Henderson et al., 2010; Guo et al., 2013; Ma et al., 2013; Batra et al., 2017; Schippers et al., 2017; Yuen et al., 2022b). Here we hypothesized that the therapeutic effects of NAc DBS may be due to its ability to rapidly modulate tonic dopamine concentrations. DBS of the VTA, the main dopaminergic afferent to the NAc, may also achieve a similar effect. In the present study, M-CSWV was utilized to elucidate the effects of high frequency stimulation (HFS) of both the VTA and the NAc on tonic dopamine levels in the nucleus accumbens core (NAcc) with or without acute cocaine administration (Yuen et al., 2021a).

## 2. Materials and methods

### 2.1. Animal subjects

Male Sprague-Dawley rats (250–300 g; Envigo, IN, USA) were used for this study. Rats were kept in social housing in an association for assessment and accreditation of laboratory animal care international (AAALAC) accredited vivarium following a standard 12-h light/dark cycle at constant temperature (21°C) and humidity (45%) with *ad libitum* food and water. The present studies were approved by the Institutional Animal Care and Use Committee (IACUC), Mayo Clinic, Rochester. The NIH Guide for the care and use of laboratory animals guidelines (Department of Health and Human Services, NIH publication No. 86-23, revised 1985) were followed for all aspects of animal care.

### 2.2. Electrode fabrication

Carbon fiber microelectrodes were fabricated using an established standardized CFM design at Mayo Clinic (Chang et al., 2013; Oh et al., 2016). Each microelectrode involved isolating and inserting a single carbon fiber (AS4, diameter = 7 μm; Hexcel, Stamford, CT, USA) into a silica tubing (20 μm ID, 90 μm OD, 10 μm coat with polyimide; Polymicro Technologies, Phoenix, AZ, USA). The connection between the carbon fiber and the silica tubing was covered with epoxy resin. The silica tubing was then attached to a nitinol extension wire (Nitinol #1, an alloy of nickel and titanium; Fort Wayne Metals, IN, USA) by a silver-based conductive paste (Chang et al., 2013). The carbon fiber attached nitinol wire was insulated with polyimide tubing (0.0089′′ ID, 0.0134′′ OD, 0.00225′′ WT; Vention Medical, Salem, NH, USA) up to the carbon fiber sensing segment. The exposed carbon fiber was trimmed under a dissecting microscope to a length of ∼50 μm. Teflon-coated silver (Ag) wire (A-M systems, Inc., Sequim, WA, USA) was prepared as an Ag/AgCl counter-reference electrode by chlorinating the exposed tip in saline with a 9 V dry cell battery. CFMs were pretested in a flow cell prior to coating deposition with a PEDOT:Nafion deposition solution (Vreeland et al., 2015), which minimized the effect of biofouling *in vivo*.

### 2.3. Implantation of recording and stimulating electrodes

Each rat was anesthetized with urethane (1.5 g/kg i.p.; Sigma-Aldrich, St Louis, MO, USA) and administered buprenorphine (0.05–0.1 mg/kg s.c., Par Pharmaceutical, Chestnut Ridge, NY, USA) for analgesia. Following anesthesia, they were placed in a stereotaxic frame (David Kopf Instruments, Tujunga, CA, USA). Respiratory rate (RespiRAT, Intuitive Measurement Systems, AZ, USA) and hind-paw and tail pinch were used to monitor the physiological state and depth of anesthesia, respectively. Using a standard rat brain atlas (Paxinos and Watson, 2007), three trephine holes were drilled, the first for placement of a CFM into the NAcc (all coordinates from bregma: AP 1.2 mm, ML 2.0 mm, DV 6.5–7.5 mm from dura), the second for a stimulating electrode into the VTA (twisted bipolar stimulating electrode–Plastics One, MS 303/2, Roanoke, VA, USA, with the tips separated by ∼1 mm; AP −5.3 mm, ML 0.9 mm, DV 7.5–9 mm from dura), and a third for an Ag/AgCl into the contralateral cortex (Figure 1; Clark et al., 2010). For NAcc stimulation experiments, a bipolar concentric simulating electrode (MicroProbes, Gaithersburg, MD, USA) was implanted immediately posterior and medial to the CFM in the NAcc (∼0.3 mm apart).

### 2.4. Recordings and stimulation parameters

The depths of the stimulating electrode in the VTA and CFM in the NAcc were first adjusted to obtain a robust stimulation-evoked dopamine signal as measured by FSCV (−0.4 to 1.3 V sweep; 10 Hz; see Supplementary Figure A1). Stimulation parameters were biphasic pulses at 60 Hz, 0.2 ms pulse width, 0.2 mA, and 2 s duration. Stimulation and FSCV were both performed using the WINCS Harmoni system (Lee et al., 2017), a wireless stimulation and neurochemical sensing system.

Once the optimal electrode depths were identified, the system was switched to the M-CSWV sensing technique (see Figure 1B). After 60 min of stabilization, either VTA or NAc biphasic pulse stimulation was applied at 130 Hz (0.2 ms, 0.2 mA) continuously. The delivered stimulation was interleaved with the M-CSWV recording to minimize artifacts. Once the signal was restabilized to a new plateau (≥ 30 min), i.v. saline (1 ml/kg) was administered as a negative control while stimulation and recording continued. After 30 min, i.v. cocaine (2 mg/kg) was administered (infused over 1 min *via* cannula at tail vein; dissolved in 0.5 ml of normal saline). After another 30 min of observation, the stimulation was turned off. Post-stimulation, the animal was observed for another 30 min before being sacrificed using Fatal-Plus injection (pentobarbital 390 mg/ml; 10 ml).

### 2.5. Pharmacological confirmation

In a separate group of animals (*N* = 5), alpha-methyl-p-tyrosine (AMPT; 250 mg/kg, i.p.), a tyrosine hydroxylase inhibitor, was given to further confirm the recording of dopamine by M-CSWV. Tyrosine hydroxylase is the rate limiting enzyme of catecholamine biosynthesis, converting tyrosine into L-DOPA, the precursor to dopamine. Thus, AMPT administration, acting as a negative control, was expected to decrease the voltammetric signal if the signal indeed arose from dopamine.

### 2.6. Calibration of electrodes

After experimentation, changes in dopamine release in individual CFMs were calibrated *in vitro* with dopamine solutions of different known concentrations. This is in a similar fashion to previously described procedures in the literature (Oh et al., 2018).

### 2.7. Histological analysis

CFM and stimulation electrode trajectories were confirmed by histological analysis. Brains were removed from euthanized animals and immersed in 4% paraformaldehyde overnight for fixation. After fixation, 60 μm coronal sections were cut on a freezing microtome. The sections were stained with cresyl violet. The location of the stimulating and CFMs were identified under light microscopy (Supplementary Material B) based on (Paxinos and Watson, 2007).

### 2.8. Statistical analysis

Statistical analysis was performed using repeated measures one-way ANOVA and two-tailed paired *t*-tests in relevant *post hoc* analyses (PRISM 8, GraphPad). For comparison, the levels were all measured by averaging over 10 data points, i.e., 10 s. In cases where i.v. drug was administered, the 10 data points centered at peak within 10 min of injection.

After ANOVA tests were performed among the positive control, negative control, VTA stimulation and NAcc stimulation groups, paired *t*-tests were used to demonstrate sequential changes in the *post hoc* analysis. In the control experiments, pre-injection baseline tonic dopamine concentrations were compared to the post-saline levels, and the post-saline levels were compared with the post-cocaine peak levels. In the NAcc stimulation experiments (see Figure 4), the initial stabilized baseline levels before injection were compared with the trough levels (not seen during VTA experiments) during stimulation. Then, similarly, the new baselines were compared with the post-saline levels, and the post-saline levels were compared with the post-cocaine peak levels. In VTA stimulation experiments (see Figure 5), the initial baselines were compared with the new baselines during stimulation. The new baselines were compared with the post-saline levels, and these post-saline levels were compared with the post-cocaine levels. All error bars and shaded areas are represented as S.E.M. statistical significance was set at *p* < 0.05. Bonferroni correction was applied in cases with multiple comparisons.

**FIGURE 2:**
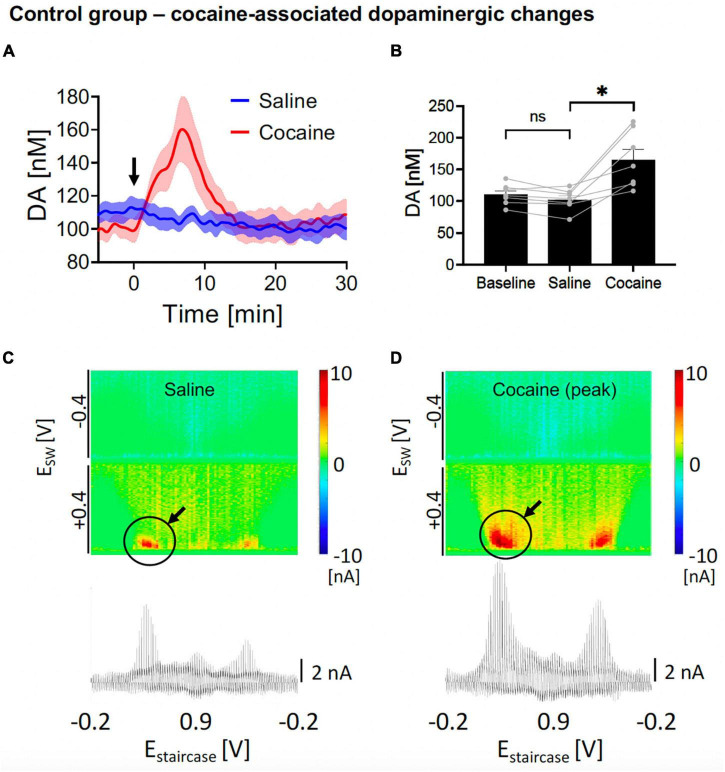
Changes in nucleus accumbens core (NAcc) tonic dopamine concentrations after saline and cocaine. **(A)** Rapid increase in dopamine was seen after i.v. cocaine administration (2 mg/kg) compared to i.v. saline (1 ml/kg). Arrow denotes time of drug administration. **(B)** Saline did not significantly alter tonic dopamine levels (–8.0 ± 3.4 nM, *N* = 7 rats, *p* = 0.054), whereas cocaine rapidly increased dopamine levels (+62.9 ± 14.9 nM, +62%, *N* = 7 rats, *p* = 0.006). Two out of seven of the sample had a stimulating electrode (turned off) adjacent to the recording electrode; both showed brisk increase in tonic dopamine levels with cocaine administration. *Denotes *p* < 0.025 (0.05/2, with Bonferroni correction, given there are two *t*-tests here). **(C,D)** Representative color plots and voltammograms after saline and cocaine administration, respectively.

**FIGURE 3:**
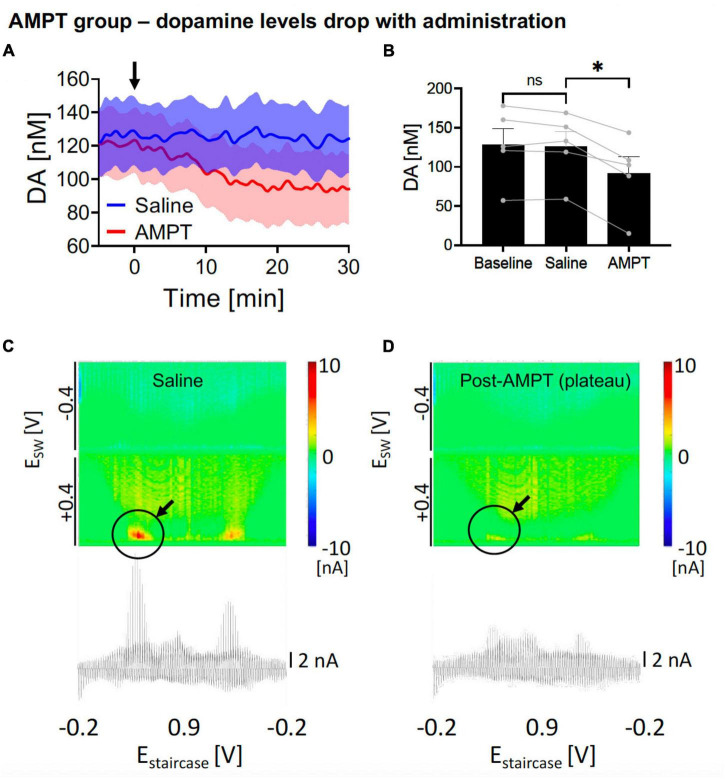
Changes in nucleus accumbens core (NAcc) tonic dopamine concentrations after saline and alpha-methyl-p-tyrosine (AMPT). **(A)** Gradual reduction in dopamine tonic levels was seen after i.p. AMPT administration (250 mg/kg) compared to i.v. saline (1 ml/kg). Arrow denotes time of drug administration. **(B)** Saline did not significantly alter tonic dopamine levels (–2.2 ± 3.1 nM, *N* = 5 rats, *p* = 0.513), whereas AMPT reduced dopamine levels (–34.5 ± 5.7 nM, –27%, *N* = 5 rats; *p* = 0.004). *Denotes *p* < 0.025 (0.05/2, with Bonferroni correction, given there are two *t*-tests here). **(C,D)** Representative color plots and voltammograms, after saline and AMPT administration, respectively.

**FIGURE 4:**
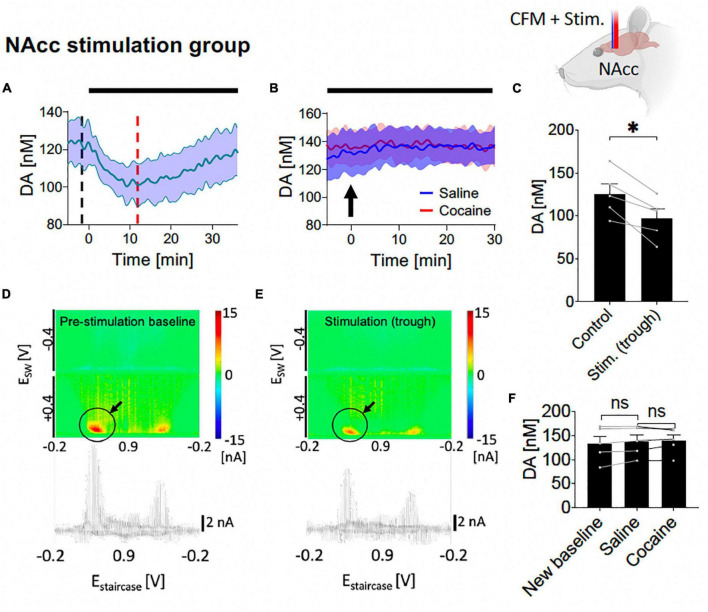
Tonic dopamine concentrations during nucleus accumbens core (NAcc) high frequency stimulation (HFS) and after cocaine administration. **(A,C)** Stimulation suppressed tonic dopamine levels (–28.3 ± 6.3 nM, –20%; *N* = 5 rats, *p* = 0.011). **(B,F)** Cocaine-induced increases in tonic dopamine levels were attenuated by stimulation to non-significant levels (new baseline vs. saline, –4.9 ± 2.6 nM, *N* = 5 rats, *p* = 0.131; saline vs. cocaine peak, 1.3 ± 3.5 nM, *p* = 0.739). Black bars represent stimulation period. Arrow denotes drug administration. *Denotes *p* < 0.017 (0.05/3, with Bonferroni correction, given there are three *t*-tests here); ns, non-statistically significant. **(D,E)** Representative color plots and voltammograms, corresponding to the time points marked by black and red dotted lines in panel **(A)**, respectively. Further trend in tonic dopamine levels after local HFS was stopped demonstrated no marked changes in levels (Supplementary Figure A2).

**FIGURE 5:**
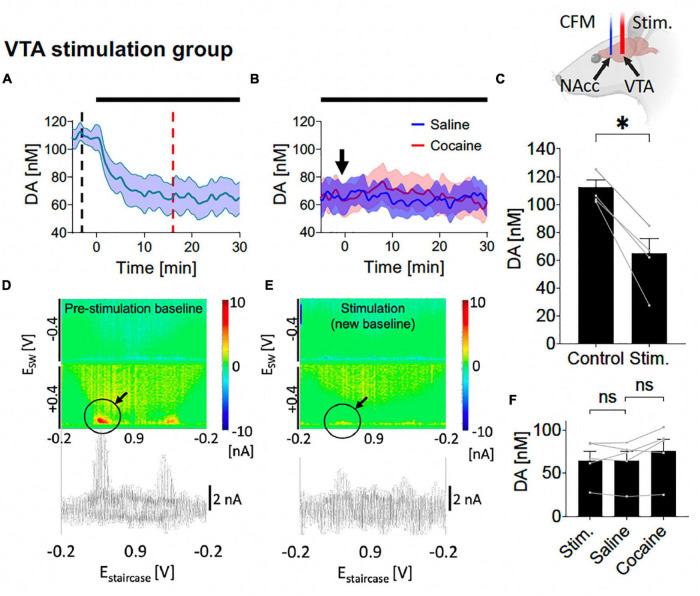
Tonic dopamine concentrations during ventral tegmental area (VTA) high frequency stimulation (HFS) and after cocaine administration. **(A,C)** Stimulation suppressed tonic dopamine levels (–47.3 ± 7.0 nM, –42%; *p* = 0.002). **(B,F)** Cocaine-induced increases in tonic dopamine levels were attenuated by stimulation to non-significant levels compared to the new baseline (+11.2 ± 5.0 nM, +17%; *p* = 0.091). Black bars represent stimulation period. Arrow denotes drug administration. *Denotes *p* < 0.017 (0.05/3, with Bonferroni correction, given there are three *t*-tests here); ns, non-statistically significant. **(D,E)** Representative color plots and voltammograms, corresponding to the time points marked by black and red dotted lines in panel **(A)**, respectively. Further trend in tonic dopamine levels after local HFS was stopped demonstrated no continued suppression in levels (Supplementary Figure A5). Representative color plot and voltammogram of new baseline after i.v. cocaine administration (2 mg/kg) during VTA stimulation is shown in Supplementary Figure A6.

## 3. Results and discussion

### 3.1. Control experiments

In the positive control experiments, after implanting the CFM at the optimal position within NAcc (see section “Materials and methods”), we elicited cocaine-induced dopamine changes by first administering i.v. saline and then i.v. cocaine, while tonic dopamine levels were recorded using M-CSWV. One-way ANOVA test among the three levels (baseline, saline, cocaine) showed significant differences (*F* = 17.95, *p* = 0.0047). In the *post hoc* analysis, as expected, saline administration did not evoke a statistically significant change in peak tonic dopamine concentration compared to pre-saline levels (*N* = 7 rats; paired *t*-test, *p* = 0.054; Figures 2A, B, blue). The dopamine levels were then observed to rapidly increase after acute i.v. cocaine administration [*N* = 7 rats; paired *t*-test, *p* = 0.022; change = +62.9 ± 14.9 nM (59%); time to peak = 6.8 ± 0.8 min; Figures 2A, B, red]. The pseudocolor plots of the peak dopamine concentration after saline administration (Figure 2C) and after cocaine administration (Figure 2D) showed clear differences in the magnitude of the dopamine oxidation current, indicating a much higher concentration present.

In the negative control experiments, AMPT, a tyrosine hydroxylase inhibitor, was applied intraperitoneally (i.p.) to reduce dopamine production. This was compared against i.p. saline. One-way ANOVA test among the three levels showed significant differences (*F* = 35.45, *p* = 0.0007). In the *post hoc* analysis, i.p. AMPT administration (250 mg/kg) did acutely reduce tonic NAcc dopamine concentrations over 30 min [*N* = 5; paired *t*-test, *p* = 0.004; change = −34.5 ± 5.7 nM (−27%); time to stable baseline = 25.3 ± 2.2 min; Figures 3A, B, red] but this was not observed in i.p. saline (*N* = 5; paired *t*-test, *p* = 0.513; Figures 3A, B, blue). This is further visualized by the pseudocolor plots, demonstrating a sharp decrease in dopamine oxidation current after 30 min of AMPT (Figure 3D), compared to after 30 min of saline (Figure 3C).

### 3.2. NAcc HFS reduces tonic NAcc dopamine levels and attenuates the effects of cocaine

Here, saline and cocaine administration was repeated on the background of NAcc stimulation (starting at least 30 min before saline administration and continued until 30 min after cocaine administration). With one-way ANOVA test, there were significant differences among the six different levels (control, trough during stimulation, new baseline during stimulation, level-post-saline, level-post-cocaine, post-stimulation) (*F* = 11.31, *p* = 0.010). With further *post hoc* analysis, NAcc HFS elicited an initial decrease in tonic dopamine concentration [*N* = 5; paired *t*-test, *p* = 0.011; change = −28.3 ± 6.3 nM (−20%); Figures 4A, C], followed by a relatively rapid return to baseline. Pseudocolor plots demonstrate a significant marked decrease in dopamine oxidation current during NAcc HFS (Figure 4E) compared to pre-stimulation baseline (Figure 4D).

Next, i.v. saline was administered (1 ml/kg) with continuous HFS, and, as before, did not significantly affect NAcc tonic dopamine concentrations over 30 min (*N* = 5; paired *t*-test, *p* = 0.131; Figures 4B, F). Thereafter, surprisingly, with continuous HFS of the NAcc, the cocaine-induced increases in tonic dopamine concentrations seen before were eliminated when i.v. cocaine was given, no longer leading to an increase compared to pre-cocaine levels (*N* = 5; paired *t*-test, *p* = 0.739; Figures 4B, F).

### 3.3. VTA HFS reduces tonic NAcc dopamine levels and attenuates the effects of cocaine

Here, saline and cocaine administration was repeated on the background of VTA stimulation (starting at least 30 min before saline administration and continued until 30 min after cocaine administration). With one-way ANOVA test, there were significant differences among the five different levels (control, new baseline during stimulation, level post-saline, level-post-cocaine, post-stimulation) (*F* = 11.28, *p* = 0.011). With further *post hoc* analysis, VTA HFS elicited a decrease in tonic dopamine concentration which persisted over the 30 min [*N* = 5; paired *t*-test, *p* = 0.002; change = −47.3 ± 7.0 nM (−42%); Figures 5A, C]. Pseudocolor plots demonstrate a significant decrease in dopamine oxidation current during VTA HFS (Figure 5E) compared to pre-stimulation baseline (Figure 5D).

Next, i.v. saline was administered (1 ml/kg) with continuous HFS, and, as before, did not significantly affect NAcc tonic dopamine concentrations over 30 min (*N* = 5; paired *t*-test, *p* = 0.943; Figures 5B, F). Thereafter, with continuous HFS of the VTA, the cocaine-induced increases in tonic dopamine concentrations seen without stimulation were eliminated, no longer leading to a statistically significant increase compared to pre-cocaine (*N* = 5; paired *t*-test, *p* = 0.091; Figures 5B, F).

### 3.4. Interpretation

The present study demonstrated that cocaine-induced increases in tonic dopamine levels in the NAcc can be attenuated by HFS of the NAcc or of the VTA. In addition, VTA HFS resulted in a persistent suppression of NAcc tonic dopamine levels.

Interestingly, there was an initial trough in the dopamine levels at the start of NAcc HFS (Figure 4 and Supplementary Figure A3), followed by a return to baseline. Previous *ex vivo* voltammetry studies have shown that both electrical and optogenetic brief stimulation of dopaminergic terminals in the NAc can lead to local phasic release of dopamine (Melchior et al., 2015). Importantly, these studies have also shown that longer duration stimulations lead to lower magnitude stimulation-induced phasic release. Phasic dopamine release is measured on the order of seconds, whereas in the current study, the time resolution of M-CSWV was every 10 s. Therefore, it is possible that there may have been an initial phasic release of dopamine which was not detected by M-CSWV. This increase in dopamine may have then led to the activation of D2 autoreceptors in the VTA and NAcc, which reduced both the release of dopamine and excitability of dopamine neurons (Wieczorek and Kruk, 1995; Ford, 2014). Together with a depletion of presynaptic dopamine vesicular stores, this may have contributed to a decrease in the tonic levels of dopamine in the NAcc. One possibility is that as the D2 autoreceptor feedback became weaker, the tonic dopamine levels stabilized to an equilibrium. However, previous studies have shown the activation time of D2 autoreceptors is of the order of subseconds to seconds (Kennedy et al., 1992; Benoit-Marand et al., 2001); whereas in the present study, the troughs took ∼10 min to reach full reduction, implying there are likely other factors at play. Another possibility is back propagation of signals from NAc to VTA but this is yet to be confirmed.

Norepinephrine is a potential electroactive interferent that could affect dopamine measurements given their similarities in reduction-oxidation characteristics. However, previous microdialysis studies show that the NAcc, which we targeted, has a relatively low concentration of norepinephrine (McKittrick and Abercrombie, 2007).

Inhibition and activation of other local neurons (e.g., glutamatergic and GABAergic) are also possible, but it is difficult to ascertain how this may interact with the dopaminergic neurons in this case. A recent voltammetry study has shown that electrical stimulation leads to multi-synaptic modulation of dopamine release, as a gamma-aminobutyric acid (GABA) antagonist increased electrical stimulation-evoked release of dopamine, compared to optogenetic stimulation, which only targeted dopaminergic terminals (Melchior et al., 2015). In contrast, microdialysis studies have shown conflicting results. In naïve rodents, HFS of the NAc did not affect dopamine and glutamate levels but increased GABA levels (Varatharajan et al., 2015). Another study which specifically stimulated the NAcc also showed no changes in dopamine levels (Van Dijk et al., 2011). However, in rats treated with morphine, NAcc HFS reduced glutamate levels (Yan et al., 2013). In a depressed rat model, there were no changes in GABA or dopamine with NAc shell stimulation (Schumacher et al., 2020). Although microdialysis can measure multiple neurochemicals, most of these studies sampled at 30-min intervals, which would not capture the trough observed here.

Both the NAcc (Liu et al., 2008; Knapp et al., 2009; Guo et al., 2013; Schippers et al., 2017) and shell (Knapp et al., 2009; Henderson et al., 2010; Ma et al., 2013; Batra et al., 2017) have been shown to be promising DBS targets for SUD for substances such as morphine, alcohol, heroin, and methamphetamine. The underlying treatment mechanism has not been fully understood. The present results suggest that one possibility is that accumbal dopamine extracellular levels are modulated by the local HFS, leading to suppression of the reward effect associated with cocaine-induced elevations in tonic dopamine levels (Schultz, 2016). Given its role as a monoamine reuptake inhibitor, cocaine normally increases dopaminergic concentration in the synapses (Sora et al., 2001). It is possible that local DBS may either alter cocaine activity at the local dopamine reuptake transporters and/or dopamine reserve, or it may reduce the ability of cocaine molecules to diffuse to these transporters due to factors such as vasoconstriction or tissue damage. Two out of seven of our control group were performed with a stimulating electrode adjacent to the recording electrode and both showed a brisk increase in tonic dopamine levels after cocaine administration, which makes tissue damage an unlikely explanation. The possibility that DBS can modify dopamine transporter (DAT) availability has been raised previously in Parkinson’s disease patients (Lokkegaard et al., 2007; Loser et al., 2021).

Other possibilities may include down regulation of active dopaminergic transporters. The diminished response is consistent with a preclinical study where DBS of the NAc (shell) increased cocaine self-administration (Kallupi et al., 2021). This may be because the cocaine-associated effect is less marked with DBS and hence the animals would need to self-administer more to attain the same elevations in tonic dopamine levels. However, more experiments are required to confirm this hypothesis.

In contrast to NAc HFS, VTA HFS led to a decrease in NAcc dopamine levels that did not recover over the course of the experiment (Figures 5A, D and Supplementary Figure A4). There are at least two possible explanations for this phenomenon. First, continuous VTA HFS may have depleted presynaptic dopamine vesicular stores in the NAcc, which may have accounted for the initial peak observed immediately upon stimulation. In turn, this would lead to a reduced tonic level until vesicular stores could be replenished by dopamine synthesis. This is consistent with a previous study showing medial forebrain bundle (MFB) stimulation could reduce the dopamine level in the NAc to 70–80% of baseline during 2 h of stimulation (Bregman et al., 2015). Amperometry studies also showed that prolonged MFB stimulation can deplete presynaptic dopamine vesicular stores in the NAc (Fielding et al., 2013). As the mesolimbic dopaminergic pathway is contained within the MFB, it is likely that MFB stimulation would involve stimulating the VTA-NAc pathway. Second, dendritic release of dopamine in the VTA has been shown to activate autoinhibitory D2 receptors (De Jong et al., 2015), resulting in reduced terminal release of dopamine in the NAc. However, this effect is expected to be short-lived, as continuous VTA HFS would also be expected to deplete dopamine dendritic stores. One other speculative cause is that VTA HFS induced a depolarization block of dopaminergic axonal firing, which appeared sustained after stimulation was discontinued. Given the tonic dopamine levels did not recover, this suggests dopaminergic dynamics may be different between the VTA and NAc, possibly from different neurochemical and autoreceptor distribution and sensitivity.

Microdialysis studies have shown that extracellular dopamine release in the NAc is regulated by GABA (inhibitory), dopamine (inhibitory), glutamate (excitatory), and acetylcholine (facilitatory) receptors in the VTA (Westerink et al., 1996; Lester et al., 2010). It is possible that the electrical stimulation could lead to overfiring of GABA neurons within the VTA as well as suppression of glutamate neurons. Further pharmacological tests may potentially facilitate confirmation of this hypothesis.

Functional magnetic resonance imaging (fMRI) in rodent and in swine models have shown that electrical stimulation of the VTA not only induced dopamine release in the NAc (phasic release as detected by FSCV) but also led to increased blood-oxygen-level-dependent (BOLD) responses (Helbing et al., 2016; Settell et al., 2017). However, the latter appeared to be glutamate-dependent (Helbing et al., 2016). This suggests that the clinical effects of VTA DBS is likely to be much more complex and involves multiple other neurotransmitter systems besides dopamine.

In addition to SUD, NAc DBS has been of great interest for application to a number of neuropsychiatric diseases, such as depression (Yuen et al., 2021b), Tourette’s syndrome (Baldermann et al., 2016), and obsessive-compulsive disorder (Denys et al., 2010). It is unknown how DBS of the NAc and its surrounding structures, such as the anterior limb of internal capsule, may treat a range of diseases with such different clinical features. Nevertheless, studies have shown that dopamine plays a role in all these diseases. Thus, DBS may possibly modulate or even re-establish the dysregulated dopaminergic signaling, leading to symptomatic improvements and reversing neuroplasticity related changes (Denys et al., 2004; Buse et al., 2013; Tye et al., 2013).

Although the VTA is vital in the expression of a number of drug-related behaviors, such as behavioral sensitization (Oliva and Wanat, 2016), VTA DBS currently has a limited role in clinical practice. However, in a small case series, high-frequency VTA DBS appeared to be an effective treatment for medically refractory cluster headache (Akram et al., 2016). Given there is evidence that dopamine levels are elevated in circulating platelets of cluster headache (and migraine) patients (D’Andrea et al., 2006), it has been suggested that cluster headache may be a consequence of overactivity of the dopaminergic and autonomic systems (D’Andrea et al., 2019). Evidence of dysfunction of dopaminergic systems is further elucidated in a study where apomorphine, a non-selective dopamine D2 receptor agonist, was given to cluster headache patients that resulted in significantly lower evoked growth hormone release compared to healthy volunteers (Lepper et al., 2013).

Optogenetic studies have provided insight into the possible behavioral effects of VTA stimulation. One rodent study demonstrated continuous (“tonic”) optogenetic stimulation of VTA dopaminergic neurons can reduce ethanol self-administration (Bass et al., 2013). In addition, other studies showed that optogenetic excitation and inhibition of VTA dopaminergic neurons can both inhibit and induce depression-like behavior, respectively (Tye et al., 2013). Although optogenetic and electrical stimulations have different underlying mechanisms of activation, one mouse study demonstrated they activate similar brain regions under certain conditions (Weidner et al., 2020).

Given the reduction in tonic dopamine levels and attenuation of the cocaine-induced response, VTA DBS may be helpful in not only treating SUD but also pathologies associated with hyperdopaminergic states such as mania, schizophrenia, and dopamine dysregulation syndrome, where excessive dopamine in the system may lead to excessive risk-taking behavior (Berk et al., 2007; Grace, 2016; Ashok et al., 2017). In addition, dopamine-containing cells in the VTA that comprise the mesolimbic dopaminergic projection are highly critical for the regulation of incentive motivation to natural and drug-related rewards (Blaha and Phillips, 1996; Schultz et al., 1997; Horvitz, 2000). In addition, by modifying the tonic level of dopamine here, it may be possible to replicate changes induced by different pharmacological agents and use it as a pathological model for other diseases such as depression. Likewise, given dopamine is also associated with non-drug reward, excessive depression of dopamine levels in a normal dopaminergic state may theoretically lead to anhedonia, anorexia, and/or depression. Therefore, one must be careful with implementing this form of intervention at the level of dopaminergic cells.

It should be noted the current study utilized anesthetized naïve rodent models with acute administration of cocaine. Larger animals and chronic addiction models will be necessary to verify the dopamine-attenuating effect of HFS. It would be useful to know the effects of HFS on models of SUD of other substances, especially those that are not psychostimulants, such as opioids, and alcohol. Also, the effect on behaviors associated with SUD, such as craving and withdrawal, needs to be explored. Further mechanistic studies such as manipulation of dopamine transporter availability and other biochemical essays are also warranted.

Previous literature also suggested there are persistent exposure of drugs does not necessarily lead to addictive behavior and dopamine is likely to be only one contributing factor to the behavioral changes observed. Therefore, one must consider the impact of other biological processes, such as changes in synaptic plasticity and other neurochemicals (e.g., serotonin) (Pascoli et al., 2011; Li et al., 2021; Yuen et al., 2022a).

## 4. Conclusion

In summary, this study elucidated the tonic dopaminergic dynamics with NAc and VTA HFS with high spatiotemporal resolution. HFS appeared to have an alleviating effect on the elevations in tonic dopamine levels associated with cocaine administration. This may explain how NAc DBS was found to be therapeutic in both preclinical models and patients suffering from SUD. Dopamine, measured by M-CSWV, may provide a useful closed-loop biomarker for DBS, given the pivotal role of dopamine in many neuropsychiatric pathologies.

## Data availability statement

The raw data supporting the conclusions of this article will be made available by the authors, without undue reservation.

## Ethics statement

This animal study was reviewed and approved by the Mayo Clinic IACUC.

## Author contributions

KL, YO, and JY conceptualized the study. JY conducted experiments, collected the data, and drafted the first manuscript. AR manufactured the 3D-printed electrode holder. JY, YO, and HS designed the analyses. JY and HS conducted the analyses. KL, HS, and YO supervised all aspects of the work. JY and YO drafted the figures. All authors critically reviewed and revised the manuscript and accepted the final version of the manuscript.
