# Supplementary material for: High frequency deep brain stimulation can mitigate the acute effects of cocaine administration on tonic dopamine levels in the rat nucleus accumbens
Source: Front Neurosci. 2023 Jan 30;17:1061578. doi: 10.3389/fnins.2023.1061578 (PMC9922701; doi:10.3389/fnins.2023.1061578)
Supplement: Supplementary file 1 [file Data_Sheet_1.docx]

Supplementary Material A – Additional color plots and voltammograms


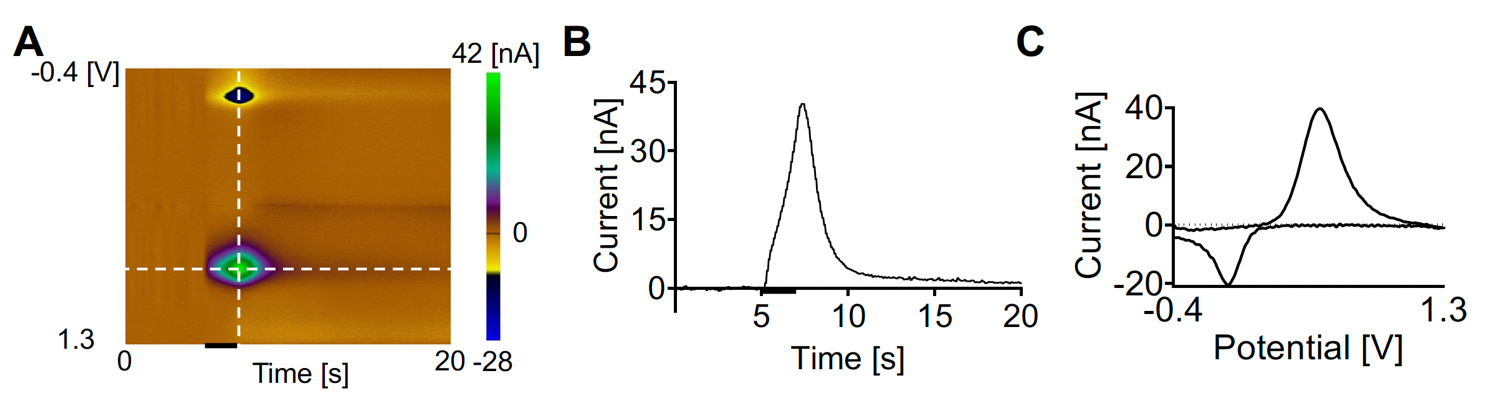


**Supplementary Figure A1.** Representative data of phasic dopamine release at nucleus accumbens core evoked by ventral tegmental area electrical stimulation (60 Hz, 0.2 ms pulse width, 0.2 mA over 2 s) detected by fast scan cyclic voltammetry (-0.4 V to 1.3 V sweep; 10 Hz). A. Color plot, B. Current-Time plot, and C. Voltammogram. White dotted lines in A. represent where the measurements were taken for B. and C. Black bar denotes stimulation period.

**Nucleus accumbens core stimulation group**

**
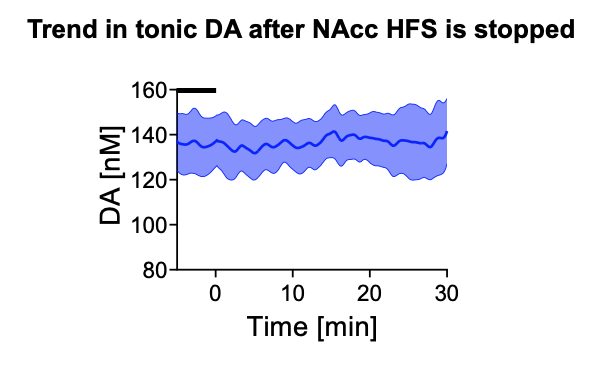
**

**Supplementary Figure A2.** Trend in tonic dopamine levels in the nucleus accumbens core after local high frequency stimulation was stopped, showing no significant changes in DA levels. Black bar denotes stimulation period. DA, dopamine.

**
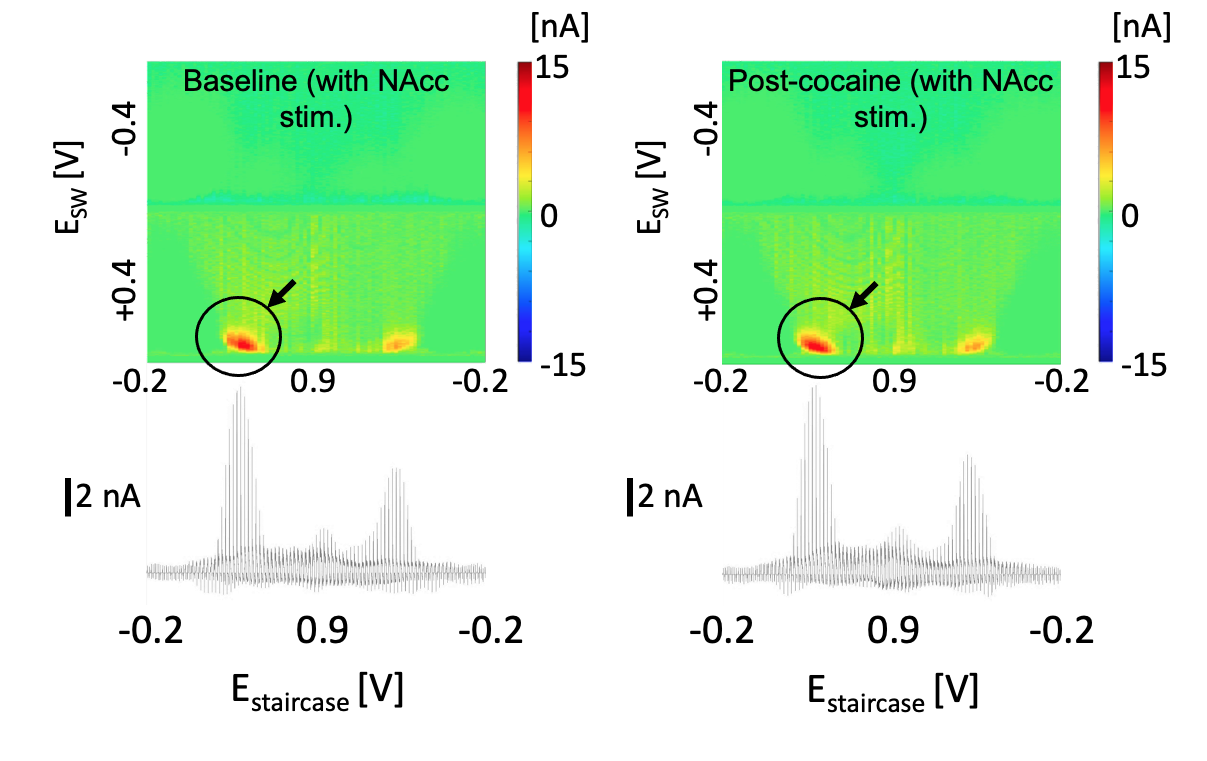
**

**Supplementary Figures A3-4.** Representative color plots and voltammograms of new baseline during nucleus accumbens core stimulation and after i.v. cocaine administration (2 mg/kg). The newly established baseline after recovery from the initial drop has a similar signal as that before the start of stimulation (see main text). Administration of cocaine did not alter the signal significantly.

**Ventral tegmental area stimulation group**

**
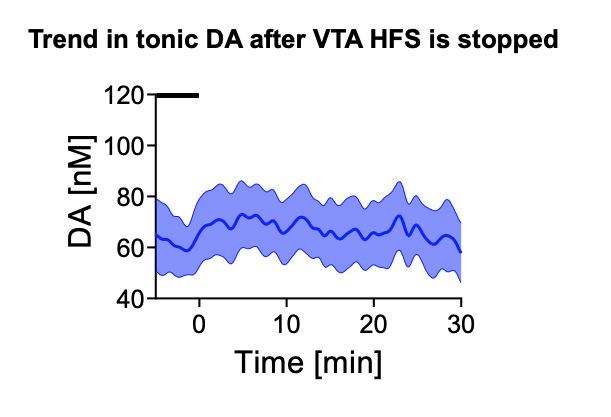
**

**Supplementary Figure A5.** Trend in tonic dopamine levels at nucleus accumbens core after ventral tegmental area high frequency stimulation was stopped, showing persistent suppression in levels. Black bar denotes stimulation period. DA, dopamine.

**
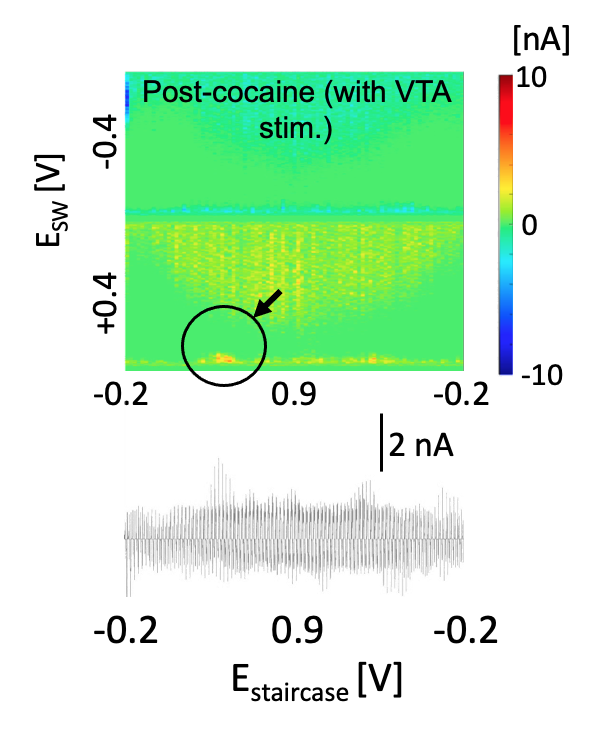
**

**Supplementary Figure A6**. Representative color plots and voltammograms of new baseline after i.v. cocaine administration (2 mg/kg) during ventral tegmental area stimulation. Administration of cocaine did not alter the signal significantly (see main text for comparison).

Supplementary Material B – Histological analysis


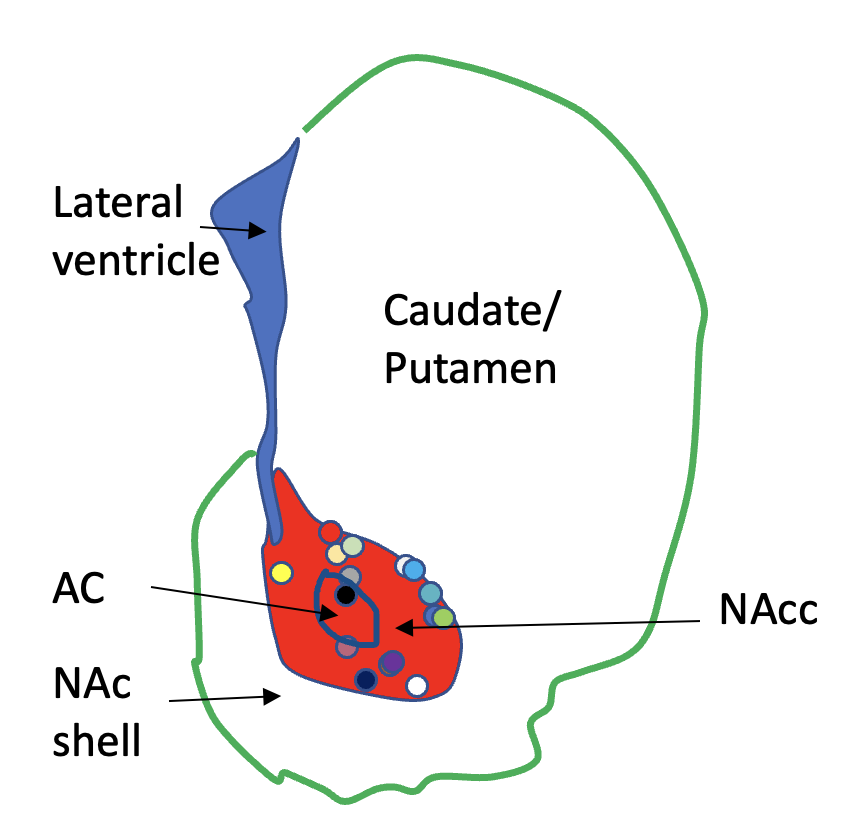

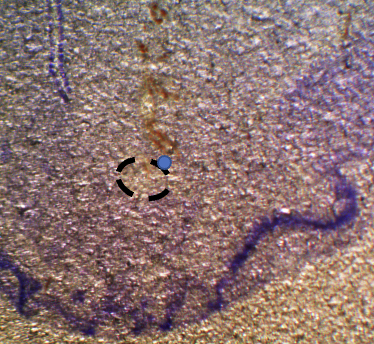


**Supplementary Figure B1.** Histological analysis of NAc CFM based on standard rat atlas at Bregma +1.2 mm (Paxinos and Watson, 2007). Right showed representative data with dotted line outlining the AC and the blue dot as the tip of the electrode. Each point is marked as a dot of different colors in the left diagram. AC, anterior commissure; NAc, nucleus accumbens; NAcc, nucleus accumbens core. N = 16.


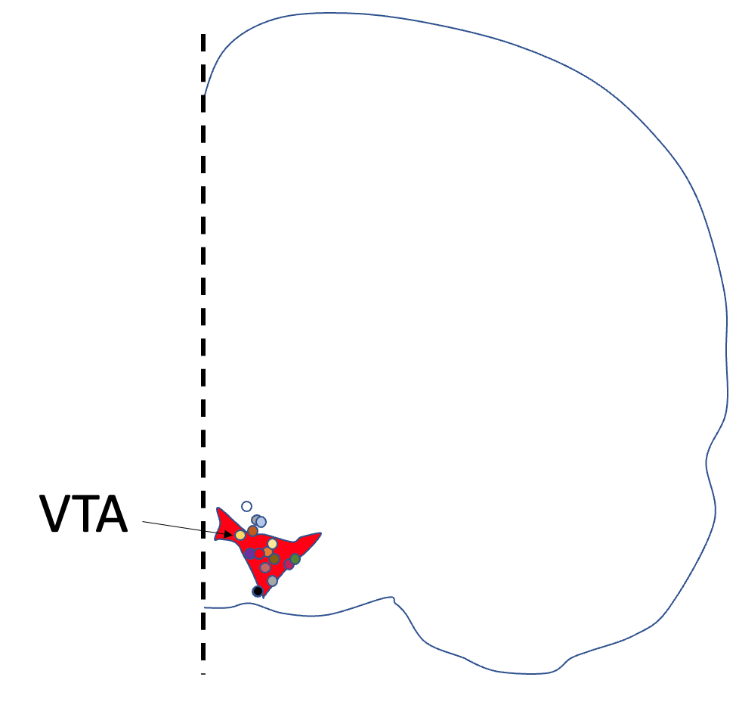

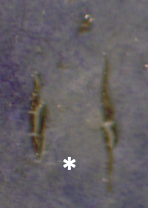


**Supplementary Figure B2.** Histological analysis of VTA stimulating electrodes based on standard rat atlas at Bregma -5.28 mm (Paxinos and Watson, 2007). Right showed representative data, magnified to show track. The * is right between the two edges of the stimulating electrodes and each point is marked as a dot of different colors in the left diagram. N = 16.
